# Biological Markers in Anxiety Disorders

**DOI:** 10.3390/jcm10081744

**Published:** 2021-04-17

**Authors:** Kacper Łoś, Napoleon Waszkiewicz

**Affiliations:** Department of Psychiatry, Medical University of Bialystok, Plac Brodowicza 1, 16-070 Choroszcz, Poland; napoleonwas@yahoo.com

**Keywords:** anxiety, biomarkers, biological markers, stress, panels

## Abstract

Anxiety disorders are one of the most commonly reported disorders in psychiatry, causing a high medical and socio-economic burden. Recently, there has been a soaring interest in the biological basis of anxiety disorders, which is reflected in an increasing number of articles related to the topic. Due to the ambiguity of the diagnosis and a large number of underdiagnosed patients, researchers are looking for laboratory tests that could facilitate the diagnosis of anxiety disorders in clinical practice and would allow for the earliest possible implementation of appropriate treatment. Such potential biomarkers may also be useable in monitoring the efficacy of pharmacological therapy for anxiety disorders. Therefore this article reviews the literature of potential biomarkers such as components of saliva, peripheral blood, cerebrospinal fluid (CSF), and neuroimaging studies. There are promising publications in the literature that can be useful. The most valuable and promising markers of saliva are cortisol, lysozyme, and α-amylase (sAA). In the blood, in turn, we can distinguish serotonin, brain-derived serum neurotrophic factor (BDNF), cortisol, and microRNA. Structural changes in the amygdala and hippocampus are promising neuroimaging markers, while in CSF, potential markers include oxytocin and 5-Hydroxyindoleacetic acid (5-HIAA). Unfortunately, research in the field of biomarkers is hampered by insufficient knowledge about the etiopathogenesis of anxiety disorders, the significant heterogeneity of anxiety disorders, frequent comorbidities, and low specificity of biomarkers. The development of appropriate biomarker panels and their assessment using new approaches may have the prospective to overcome the above-mentioned obstacles.

## 1. Introduction

**Figure 1:**
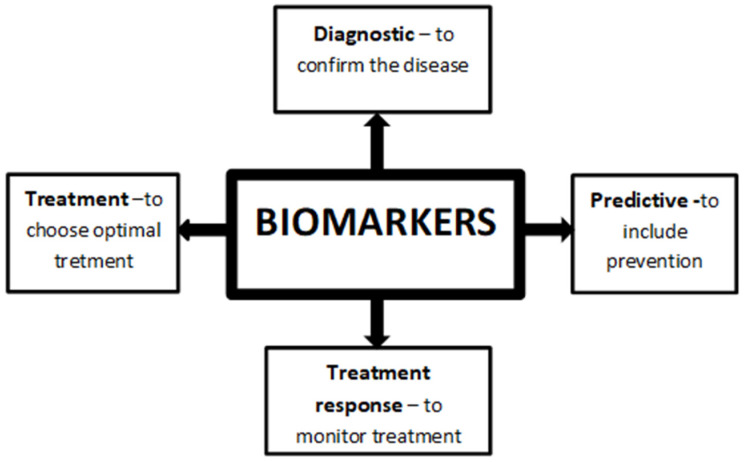
Application of biomarkers.

Anxiety disorders are among the most commonly reported mental disorders [1,2]. The literature review by Remes et al. on the epidemiology of anxiety disorders prevalence reported a worldwide spread of 3.8% to 25% [1]. Every year, in the European Union, at least 60 million people suffer from these conditions [3]. Due to the high incidence of these mental disorders, many countries are struggling with the high cost of treatment [1,4]. The annual cost in the United States is estimated at $42.3 billion [5]. Stress and anxiety cause physiological changes, in which hormone levels are altered by the activation of the hypothalamic-pituitary-adrenal (HPA) axis and the autonomic nervous system (ANS), which are especially noticeable in chronic anxiety symptoms [6,7]. The moment when the level of stress becomes disturbed is a very individual factor influenced by various circumstances [8]. It is associated with the difficulty in diagnosing and identifying disease thresholds [2]. Research shows that among the commonly conducted questionnaires, anxiety disorders are not identified in up to 50% of affected people [9]. Significant underdiagnosis and difficulties in the treatment of these disorders have been demonstrated over the years [10]. One of the modern approaches to the issue that will facilitate diagnosis and allow a better understanding of the disease is the identification of biomarkers that underlie the pathogenesis of anxiety disorders [10]. Recently there has been a trend to categorize mental disorders on the basis of objective factors such as biological markers [11,12,13]. Biomarkers are described as a trait that is accurately measured and assessed as an indicator of regular biological processes, pathological processes, or biological responses to therapeutic interventions [12,13]. Researchers have noted that markers could explain the etiology of mental illness, help to confirm diagnoses, help with the identification of susceptible people, and determine the severity of patient disease [11,14,15]. Some authors also suggested that markers could be used to adjust the treatment method to a specific patient’s case and to monitor their clinical response [11,14,15] presented in (Figure 1)Obviously, the markers should have a satisfactory level of sensitivity, specificity, and prognostic value to be used for this purpose [15].

## 2. Aim and Methods

**Figure 2:**
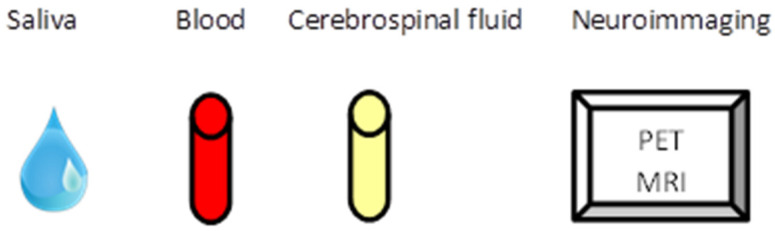
How measure potential biomarkers can be measured.

In this article, the biomarkers of anxiety disorders that may be helpful in the early diagnosis of anxiety disorders will be reviewed. In particular will be discussed those biomarkers that can be tested in saliva, plasma, CSF, and neuroimaging (Figure 2). The acquired knowledge could result in the improvement of care for patients suffering from anxiety disorders, speed up their treatment and improve their detection [10,16].

The literature review was performed using PubMed, Scopus, Google Scholar using keywords: anxiety disorders, biomarker, saliva, peripheral blood, cerebrospinal fluid, neuroimaging, and various combinations of these keywords. Valid articles were then included with the intention of covering the broadest possible range of potential markers for anxiety disorders.

## 3. Salivary

The use of saliva in laboratory diagnostics seems to be more and more popular due to its low cost and non-invasiveness [17,18,19]. Saliva contains many substances, the concentration of which exposes the wellness of the whole body, that can be used for easy and rapid detection of primary pathological symptoms in humans [17,20,21,22,23]. Saliva components can be controlled by specific and sensitive immunological and biochemical techniques, such as radioimmunoassay (RIA), enzyme immunoassay (ELISA), spectrophotometry, or chromatography [24]. On the other hand, the composition of saliva can be affected by many issues, such as circadian rhythm on secretion, age, sex, smoking, diet, and medications [25]. There are publications in the literature showing a relationship between anxiety and the level of cortisol, immunoglobulin A, lysozyme, melatonin, alpha-amylase, chromogranin A, and fibroblast growth factor 2 in saliva [26].

Cortisol is a hormone that activates metabolism, and activates the bodies “fight or flight” response, and enhances the action of other “stress hormones” such as adrenaline and noradrenaline [27,28]. Therefore, cortisol is one of the substances that are commonly used as a stress biomarker [29,30,31,32]. Miller et al., in their meta-analysis, confirmed the significance of cortisol in saliva as a biomarker for acute stress. Among the surveyed people, the authors of the article showed a decrease in morning salivary cortisol levels along with a decrease in the intensity of the stressor [33]. However, according to the constantly high level of stress, the human body is exposed to regularly high levels of cortisol. Consequently, there is a theory that after prolonged exposure to stress, the HPA axis becomes less sensitive, which affects exhaustion and consequently a decrease in the release of cortisol by the adrenal glands [34,35]. This concept has been validated by the results of a study using participants with long-term anxiety disorders, which had lower cortisol production than in healthy controls [36]. The use of cortisol in saliva as a potential biomarker of anxiety disorders requires further research, as a meta-analysis revealed high heterogeneity [26]. The study found that these results appear to be due to the lack of standardized laboratory kits and the clinical variety of enrolled participants [37].

Immunoglobulins are proteins that are responsible for specific human immune system responses. Anxiety disorders can deteriorate the immune system, and therefore the production of immunoglobulins is reduced [38]. IgA is a class of antibodies that occurs mainly in mucous membranes, including the oral mucosa. These antibodies are often the body’s primary response factor when encountering a pathogen or allergen [38]. Psychological factors can also alter the concentration of IgA in saliva; for example, a positive mood causes an increased level, while negative, stressful stimuli result in a decrease [38,39]. Studies have shown that there is a potent association between perceived stress, anxiety, and low levels of salivary immunoglobulin A [40].

Lysozyme is a protein that is continuously produced and released by monocytes and macrophages; therefore, it is commonly circulated in body tissues and secretions, including saliva. Lysozyme provides saliva antibacterial properties and contributes to antiviral defense [41,42]. Perera et al. found noticeably lower concentrations of lysozyme in saliva samples taken from academics prior to an exam, in contrast to the values after the exam [43], suggesting that salivary lysozyme is useful as a potential stress marker. Other researchers have also shown a negative association between lysozyme concentration and exposure to stress [44,45,46]. However, there is a lack of information on the relationship between lysozyme concentration in saliva and anxiety disorders.

Melatonin is a derivative of serotonin that modulates sleep phases and impacts sleep quality [47]. Ito et al. showed that melatonin concentration in saliva during sleep was correlated with anxiety disorders [48]. Interestingly, in the case of depression, this correlation was much stronger. Moreover, Paul et al. showed a significant reduction in the level of melatonin in the saliva of soldiers with post-traumatic stress disorder (PTSD) [49]. The results released by scientists seem to be extremely propitious; therefore, it is worth expanding the scientific research on melatonin because melatonin in saliva could be a valuable biomarker in the future [50].

Salivary alpha-amylase (sAA) is one of the digestive enzymes found in the oral cavity, which is responsible for both the hydrolysis of starch and glycogen as well as immunological function, providing the oral cavity protection against infections [51]. Moreover, salivary alpha-amylase has also been found to be a marker for response to incentives that activate the sympathetic system [52]. In response to stress, there is a rapid increase in the concentration of alpha-amylase in saliva, which could make it a significant biomarker in the future [53]. Jafari et al. confirmed the usefulness of sAA as a biomarker indicating its objectivity and reliability in measuring anxiety related to dental treatment [54]. Acute stress activates the axis of the sympathetic nervous system, the adrenal medulla, which is reflected both in the level of salivary alpha-amylase and in the concentration of chromogranin A in saliva [40].

Chromogranin A (CgA) belongs to the group of acid proteins that contain oligosaccharide chains, which are released from the adrenal medulla and sympathetic nerve endings, which can be detected in saliva samples [55,56]. A noteworthy increase in the absorption of CgA was found in the saliva of animals that were exposed to a stressful situation. Salivary CgA has been suggested by many researchers as a promising, sensitive biomarker in saliva for psychological stress in patients [57,58,59,60,61,62,63]. On the other hand, an important advantage of CgA determination in saliva is the independence of levels from the time of day, which makes this biomarker very promising [58].

Fibroblast Growth Factor 2 (FGF-2) is a mitogen for different kinds of cells found in saliva that is involved in physiological functions related to stress regulation and neuroregeneration [64,65]. During studies on rats, researchers proved that FGF-2 is a promising biological marker for susceptibility to stress and anxiety disorders [66]. Interestingly, the expression of fear, measured among a large group of healthy people who were subjected to stressors, correlated negatively with the level of FGF-2 measured in saliva [67]. Therefore, FGF-2 could be used as a stress biomarker. Moreover, people with a lower starting point of FGF-2 due to stress exposure could have psychological difficulties in coping with stress and, as a result, be more prone to anxiety disorders [65].

## 4. Peripheral Blood

Peripheral blood assessment is the most commonly used clinical test for detecting many diseases and is often part of complete body function assessments. Measurement of peripheral serotonergic parameters related to 5-hydroxytryptamine (5-HT, serotonin) such as whole blood serotonin, platelet serotonin transporters, and platelets inositol 1,4,5-trisphosphate (IP3) have been identified as clinical predictors of obsessive-compulsive disorder (OCD) [29]. Delorme et al. reported that a higher concentration of serotonin in whole blood was a factor that may predict better improvement in patients with OCD [68]. So far, studies related to biomarkers have shown decreased serotonin binding by platelets in patients with generalized anxiety disorders (GADs) [69], but unchanged 5-HT binding in the lymphocytes of these patients compared to controls [70].

Platelet markers such as mean platelet volume (MPV) and platelet count (PLT) reflect central serotonergic functions and are thought to reflect the serotonergic functions of the brain [71]. Ransing et al. suggested that platelet and red blood cells (RBC) markers may demonstrate to be useful etiological and predictive markers in patients with panic disorders [72]. Interestingly, research data show that stress increases platelet activity, reactivity, and immunomodulatory capacity [71]. In a 6-month study, it was shown that patients suffering from panic disorders had an elevated platelet distribution width (PDW) and red cell distribution width (RDW). However, the clinical utility of these platelet markers is not yet fully established in psychiatry [71,73,74].

It was also found that lower levels of brain-derived serum neurotrophic factor (BDNF) occurred in patients with panic disorders, further suggesting that BDNF may contribute to the therapeutic response in panic disorders [75], which was confirmed by research by Suliman et al., who confirmed significantly lower levels of brain-derived neurotrophic factor (BDNF) in the plasma of people suffering from GAD compared to the control group. In addition, clinically significant improvements were observed with paroxetine treatment reflecting restoration of BDNF levels, suggesting its potential as a biomarker [76].

Neuropeptide S (NPS) is involved in states related to fear and stress and the accompanying neuroendocrine processes [77]. Results from Jüngling et al. confirmed that the levels of NPS measured in plasma were associated with the severity of anxiety in GAD and could be considered a candidate marker for the identification of GAD. Moreover, it has also been associated with other neurochemical processes, including activation of the HPA axis and modulation of proinflammatory cytokines, neuroendocrine systems related to anxiety disorders [78,79,80].

HPA axis activation studies of panic disorders have used cortisol secretion as an indicator of HPA function through panic attacks and compared patients with panic disorders (PDs) to a control group [81]. Studies report inconsistent results, although some evidence points to higher cortisol secretion in people being tested with a PD compared to controls; other studies have found comparable cortisol levels between these two groups [29,82,83,84,85]. As indicated by Bandelow et al. [86], it is not clear whether dysfunction of the HPA axis is a potential reason for PD or a consequence of constant stress caused by recurrent panic attacks. However, HPA axis dysregulation can be considered a prognostic biomarker because higher cortisol secretion predicts worse long-term outcomes in patients with panic disorders [87,88]. Unfortunately, predictive PD biomarkers are still unclear due to inconsistent results. Social anxiety is characterized by an increased response to cortisol and lower testosterone levels [89]; Bandelow et al. [82] and Fisher et al. [90] reported that basal cortisol levels did not predict response to psychological therapy. In contrast, Petrowski et al. [91] observed evidence of HPA axis hyporeactivity in patients with a social phobia with low blood cortisol. It has been suggested that this decreased HPA axis reactivity may be related to the inability to induce adequate hormone release as a direct result of prolonged, repeated exposure to stress.

Persistent anxiety and the associated chronic stress cause proinflammatory changes that are directly related to the hypothalamic-pituitary (HPA) axis, thus, increasing the risk of excessive systemic inflammation [92]. Cross-sectional analyses have shown some indications for higher levels of IL-6 and TNF-α in people with GAD compared to those without GAD, although most studies had small sample sizes and did not sufficiently take into account confounding factors. In addition, decreased levels of adiponectin (polypeptide hormone produced and secreted into the blood with anti-inflammatory activity) were observed over time in people with GAD compared to those without GAD [93]. Relevant to mood and anxiety disorders, inflammatory biomarkers such as inflammatory cytokines and acute-phase proteins are substantially elevated in a significant proportion of patients with anxiety disorders and post-traumatic stress disorder (PTSD) [94,95]. Numerous scientific studies have shown that peripheral inflammation targets brain structures related to mood disorders and anxiety, which may be related to the effects of cytokines on neurotransmitters such as monoamines, especially dopamine (DA), as well as glutamate [94]. Considering the availability of DA, concentrations of phenylalanine and tyrosine can be found in the peripheral blood and in the cerebrospinal fluid and could serve as indirect biomarkers for the capacity to synthesize DA [96,97,98]. Inflammatory biomarkers, such as inflammatory cytokines and acute-phase proteins, are substantially elevated in a significant proportion of patients with anxiety disorders and PTSD and may be a causative agent of behavioral symptoms [94]. It could be explained by the existence of specific biological mediators between stress and inflammation, including corticotrophin-releasing factor [2]. Another inflammatory marker, C-reactive protein, has been shown to be significantly higher in men with anxiety disorders than in men without, even taking into account other disease factors and lifestyle [99]. In addition, stress and anxiety can cause physiological changes that are especially strong in chronic anxiety symptoms [100]. Thus, the presence of anxiety, especially in the long term, can cause a cascade of physiological changes, putting the individual at risk for general health conditions. Costello et al., in a recent systematic review and meta-analysis of peripheral proinflammatory cytokines in people with GAD, found that some cytokines were elevated in people with GAD compared to controls [101]. These immune factors included the CRP protein [102,103]. Another study measuring peripheral cytokine levels in a small group of GAD patients showed an increase in plasma levels of interleukin (IL)-1 and melanocyte-stimulating hormone (α-MSII) but ruled out significant variability in IL-2 [104]. However, only the CRP data were statistically significant in the meta-analysis [95].

Accumulating evidence suggests that a significant proportion of patients with anxiety-related disorders are characterized by low-grade chronic inflammation as measured by augmented peripheral and central inflammatory cytokines and other mediators of inflammatory and acute phase proteins [105,106,107]. Blood inflammatory cytokines, e.g., IL-1, IL-6, and tumor necrosis factor (TNF), their specific receptors, and acute phase reagents such as C-reactive protein (CRP), were increased in patients with anxiety [107,108].

Berardis et al. focused on the common problem of alexithymia among patients with OCD which coexists in 30% to 40% of people suffering from this disease [108]. Alexithymia is a difficulty in recognizing and managing emotions, which is associated with an increased risk of suicide among these patients. Researchers confirmed increased suicide ideations in a group of 79 patients suffering from OCD, which, interestingly, was associated with lower levels of high-density lipoprotein cholesterol (HDL-C) measured in the serum. This is probably due to the fact that patients suffering from anxiety disorders have an increased level of oxidative stress, which directly affects the oxidation (peroxidation) of lipids [109]. What is more, studies on 70 people suffering from GAD with alexithymia disorder also confirmed dysregulated cholesterol levels; in addition, researchers found variability in CRP levels among these patients, confirmed by the elevated level of inflammatory factors in the serum of people suffering from anxiety disorders [110]. Research on patients suffering from PTSD with accompanying alexithymia is also extremely interesting. Although there was a greater level of difficulty in recognizing feelings and an increase in suicidal ideation among people with PTSD, which explains the accompanying alexithymia, there was no correlation observed with the level of homocysteine measured in the serum of these patients [111]. The above relationships indicate a promising direction for future researches.

MicroRNAs (miRNAs) are regulators of gene expression that play an important role in neuronal development, in particular in the formation and shaping of synapses. Gene expression, in turn, is directly related to the neurobiological system that underlies stress and anxiety management [112]. Incorrect expression of microRNAs has been implicated in a wide variety of fear and anxiety disorders. It has also been shown that experimental regulation of potential microRNAs in the nervous system during anxiety in animals can directly influence anxiety-related behavior. Murphy et el. found individual microRNAs that were associated with the regulation of anxiety, including miR-15a, miR-17-92, miR-34, miR-101, miR-124, miR-135, and miR-155 [113]. Moreover, it has been found that both drug therapy and non-pharmacological intervention can influence the regulation of microRNAs in specific regions of the brain. This is particularly important as it offers particular hope for deepening our understanding of the underlying mechanisms of anxiety disorders as well as opens the door for new treatment strategies in the future.

Due to the relatively simple method of sampling, blood seems to be a rational source of metabolic measurements. However, because of the existence of the blood-brain barrier, drawing conclusions from the neurochemical composition of plasma about the processes taking place in the brain is not always straightforward [114]. The prevalence of anxiety disorders and the small amount of research indicates the need for further investigations of such potentially valuable approaches [115].

## 5. Cerebrospinal Fluid

Cerebrospinal fluid (CSF) is among the potential body fluids in which biomarkers may be detected. The CSF study has brought significant benefits in understanding the pathophysiology of brain disorders [116]. However, it should be taken into account that lumbar puncture is an invasive procedure, and the components of the cerebrospinal fluid do not accurately reflect the neurochemistry of the cells in the brain [117]. Therefore, this procedure is not performed very often for psychiatric disorders, and most of the data collected concerns affective or psychotic disorders, but not anxiety disorders [118].

5-Hydroxyindoleacetic acid (5-HIAA) is one of the main serotonin metabolites, and it is used to determine serotonin levels in the whole body. Interestingly in patients with major depressive disorder (MDD) and coexisting PD, a significant increase in the concentration of 5-HIAA in CSF was found equated to patients without PDA and a control group [119]. On the other hand, it was found that 5-HIAA in CSF was reduced in patients with a positive response to tricyclic drugs [120].

Neuropeptides, which are small protein-like molecules created and excreted by neurons [121,122], underlie the pathophysiology of anxiety disorders. Accordingly, they have a potential role as biological markers [123,124]. The most important neuropeptides that play a role in modulating stress and anxiety-related behavior are cholecystokinin (CCK) [125,126], oxytocin (OXT) [127,128,129,130], and ghrelin [131].

CCK seems to be one of the most plenteous neuropeptides in the brain. What is more, CCK-B receptors could be located in high density in the hypothalamus, limbic system, basal ganglia, hippocampus, cortex, and brainstem. Numerous studies have investigated the role of CCK in moderating anxiety and the stress response in humans [125,132]. Lydiard et al. showed that compared to the control group, patients with PD had lower concentrations of cholecystokinin-8 (CCK-8) in the cerebrospinal fluid [133].

OXT is a neuropeptide that is produced by the hypothalamus, which regulates the activity of many brain structures, including the amygdala, hippocampus, and cingulate cortex [134]. Additionally, OXT has various significant peripheral roles, especially in muscle contraction during labor and milk secretion [135]. The activity of OXT plays a key role in societal bearing, anxiety, mood control, and stress modulation [136]. Myers et al. confirmed that OXT plays a role in the pathophysiology of anxiety disorders [137]. OXT concentrations in the cerebrospinal fluid were significantly higher than in the plasma, and patients with higher anxiety scores had lower CSF OXT concentrations than controls [129]. These results suggest the notion that OXT concentrations may have clinical significance as an anxiety biomarker.

Ghrelin is a neuropeptide involved mainly in food intake, which additionally influences the regulation of emotions, mood, and anxiety [131]. Several studies have found that ghrelin induces anxiety effects [138], and increased ghrelin secretion under stressful conditions determines anxiety behavior and the activation of the HPA axis [139].

The level of these neuropeptides could be observed in both cerebrospinal fluid and plasma samples, suggesting their potential role as peripheral biomarkers, but due to the small research samples and the ambiguity of the results, this topic requires further study.

## 6. Neuroimaging

There is currently a growing interest in measuring microglia activation, which occurs in patients with anxiety disorders, by using neuroimaging strategies such as positron emission tomography (PET) or magnetic resonance imaging (MRI). These strategies aim to understand the role of CNS inflammation in psychiatric disorders and to be able to determine if anti-inflammatory therapies can reduce inflammation in the brain.

The effect of systemic inflammation on the brain involves glutamatergic and dopaminergic pathways that can lead to psychiatric disorders, including anxiety disorders. These pathways regulate the patient’s motivation and motor activity, as well as sensitivity to danger [137]. We can use MRI and PET imaging to assess the effects of inflammation on neurotransmitters and neurological circuits related to the reward and anxiety pathways in the central nervous system (CNS). These could serve as biomarkers for the brain’s response to treatment, and in the future, could be used as a method for studies investigating blocking or reversing inflammation in the brain, thereby more effectively detecting and treating patients with anxiety disorders [140].

One of the basic strategies for this approach is to develop radioligands that, when activated, bind to macrophages and microglia in the brain, increasing the surface expression of the translocator protein (TSPO) [141]. PET ligands that bind to TSPO are used as potential markers of activated microglia, e.g., (^11^C)-PK 11195, which exhibits an increased non-displacement binding potential (NDBP) [142]. On the other hand, there are doubts about measuring microglia activation by PET imaging. It is known that even when at rest, microglia play various important functions that cannot be ignored, for example, the sentinel-type function [141]. Moreover, microglia show graded activation responses [143], and an increase in some activation markers, such as TSPO, may not be indicative of a pure inflammatory phenotype, making the interpretation of TSPO expression difficult [144,145].

Among patients with anxiety disorders, there is an accumulation of peripheral immune cells in the perivascular and meningeal compartments, which is associated with local specific activation of microglia. Peripheral inflammatory cytokines can enter the CNS to initiate a local immune response [146,147]. In response to this chemokine, activated monocytes/macrophages travel to the brain, and it has been shown that this may contribute to behavioral changes in stress-induced anxiety behavior patterns in rodents [147,148,149]. Of particular interest in anxiety-related disorders that are directly related to inflammation in the body is the effect of inflammatory cytokines on specific areas of the brain, especially those involved in the detection of fear, anxiety, and danger. These structures include the amygdala, insula, the medial prefrontal cortex, and the anterior cingulate cortex (ACC) [94].

The amygdala is the major brain region responsible for anxiety [150]. Neuroimaging results in humans indicate that increased levels of inflammatory cytokines increase the activity of the amygdala and enlarge the amygdala [151,152,153]. Increased levels of IL-6 and TNF following administration of endotoxin to healthy people have been shown to increase the activity of the amygdala in response to socially threatening factors, which was associated with an increased sense of social separation [152]. Stress, increased amygdala neuronal activity in response to a psychosocial laboratory stressor have been related to superior stress-induced increases in IL-6 [153]. Consequently, the greater sensitivity of the amygdala to stress can lead to increased production of inflammatory cytokines, which in turn can affect the activity of the amygdala, creating a feedback effect that is linked to anxiety and its symptoms. The medial prefrontal cortex and the medial frontal gyrus are strongly connected to the amygdala and are believed to be involved in fear extinction and emotional regulation in PTSD [150,154]. Numerous studies have described an association between peripheral inflammatory cytokines and the activity of the medial prefrontal cortex under stress [151,155]. Certainly, administration of the typhoid vaccine to healthy controls persuaded mood changes that are related to increased activity in the subgenual anterior cingulate cortex (subgenual ACC) during the implicit task of emotional facial perception. Increased neuronal activity in the amygdala was correlated with an increased response of IL-6 to a psychosocial laboratory stressor, and functional connectivity analyzes showed that individuals who showed an increased inflammatory response to the stressor showed a stronger coupling between the amygdala and the dorsomedial part of the prefrontal cortex [153]. In a separate study of women undergoing chronic emotional stress related to grief, it was shown that elevated levels of IL-1beta and the soluble TNF II receptor in saliva positively correlated with the degree of ventral prefrontal activation (including subgenual ACC and orbitofrontal cortex) during grief [28,155], suggesting a link between stress, inflammation, and activation of the medial prefrontal cortex that may be important for emotional processing in stress and anxiety disorders.

Another region associated with the activity of the amygdala resulting from anxiety disorders is the insula [156,157]. For example, increased activation of the anterior amygdala and anterior insula has been observed in women with violent post-traumatic stress disorder compared to those with happy faces while matching to “fearful vs. happy target faces” [158]. It has been shown that increased sensitivity of the insula to peripheral inflammatory cytokines, especially in the presence of emotional stimuli, may alter the neural circuits, including the amygdala, medial prefrontal cortex, and ACC, inducing symptoms of anxiety, restlessness, and emotional disturbance.

A place in the CNS that is particularly influenced by inflammatory mediators in the form of cytokines is the dorsal part of the ACC (dACC). The dACC has been found to be involved with social discomfort and hence has been proposed to contain a neural “alarm system” that detects and reacts to any stimuli coming from the environment that are recognized as dangerous [159]. Further activation of the autonomic excitation system by the dACC is another element of this “human protection”, which can both identify and react to the threat cognitively, emotionally, and physically [160]. Increased dorsal ACC activity has been proposed as a mediator of hyperactivity symptoms in PTSD [161] as well as a potential family risk factor for the development of PTSD [162]. PET has shown that individuals with PTSD have increased metabolic activity in the dACC [163]. Increased activation of dACC has also been found in people with severe anxiety and obsessive-compulsive disorder [164,165,166].

Taken together, these data indicate that cytokines may increase dACC reactivity, possibly through their effects on glutamate, and thus increase sensitivity. This increased sensitivity of the dACC to the presence of cytokines may further contribute to the symptoms of PTSD anxiety and stress disorders and in trauma patients with increased inflammation [94]. Neuroimaging effects of inflammation on reward and threat circuits are particularly expressed in the amygdala, insula, and dACC areas and could be used as inflammation biomarkers. This may be especially important for the future improvement of new therapeutic strategies to better treat mood and anxiety disorders resulting from severe anxiety-related inflammation [94].

## 7. Questionnaires

We cannot forget about screening questionnaires which are important diagnostic aids. The tests are useful methods that facilitate the diagnosis of anxiety disorders. This is a very extensive topic that needs to be discussed in a separate review. However, the authors of this article would like to mention a couple of questionnaires. The occurrence of anxiety symptoms can be assessed using the Questionnaire Symptom Checklist (SCL-90) [167]. The examination allows determining the severity of symptoms in the following dimensions: somatization, anxiety, obsessive-compulsive behavior, hostility, interpersonal sensitivity, and anxiety in the form of phobias [168,169]. In turn, the self-assessment tool Perceived Stress Scale (PSS-10) can be used to assess the degree to which experienced situations are perceived by patients as stressful [170]. It concerns various subjective feelings related to problems and personal events, behaviors, and methods of coping with stress. It is used to measure the intensity of stress related to one’s own life situation in the last month [169,171]. Another questionnaire worthy of attention is the Patient Health Questionnaire for Depression and Anxiety (PHQ-4). The PHQ-4 questionnaire can be used with patients to detect people with inappropriate psychological conditions. Renovanz et al. examined patients with intracranial tumors and detected those with relevant psychological comorbidities with a sensitivity of 76.8% [172]. The Generalized Anxiety Disorder Screener (GAD-7) and the Hospital and Depression Scale (HADS-A) are used with adequate diagnostic accuracy as screening tools for generalized anxiety disorder [173,174]. The State-trait anxiety inventory (STAI) is an appropriate questionnaire to measure the self-reported presence and severity of current anxiety symptoms and generalized anxiety tendencies [174].

## 8. Discussion

Anxiety disorders are a multi-dimensional topic, as they have multifactorial origins [175], and it is unlikely that a single biomarker could explain the dynamic nature of the psychiatric illness [15]. An approach that includes both psychiatric diagnoses, taking into account the course of the disease, and a combination of various biomarkers, seems to be the most reliable [12]. Biomarkers can be used for early detection of mental states, especially those requiring urgent medical intervention, known as trait markers [176]. The most promising biological trait markers included in this article are sAA, CgA, FGF-2, NPS, and ghrelin. Another interesting group of biological markers are state markers; they show the level of clinical symptoms that can be observed in patients. It is particularly important in the case of monitoring the treatment progress and possible modification of treatment of patients suffering from anxiety disorders [176]. Such biomarkers mentioned in this review are melatonin, BDNF, 5-HIAA, microRNAs, and neuroimaging biomarkers. Numerous biomarkers, such as serotonin, cortisol, lysozyme, and inflammatory biomarkers, can perform both functions. Depending on the duration of the disease, their concentration in the body varies. Cortisol in the initial stage is elevated, which makes it possible to treat it as a trait biomarker, then its concentration decreases; therefore, we can monitor it to assess the progress of the disease. The worth emphasizing topic is the mutual relationship among biomarkers derived from different sources. The condition and functioning of the brain and its processes are best reflected by CSF. The inflammation in the brain in the course of anxiety disorders is reflected both in the results of the measurement of inflammatory factors in the blood and in the local activation of microglia in imaging tests. However, due to the existence of the blood-brain barrier, the neurochemical composition of plasma may be different from that of the CSF, and therefore it is difficult to draw direct conclusions. For example concentration of OXT in the cerebrospinal fluid is definitely higher than in the blood. In contrast, the identified microRNAs are associated with the characteristic expression of BDNF. Moreover, it is correlated with the HPA axis and the stress regulation function, which in connection with PET neuroimaging is a very promising prognosis. Given the accumulated medical knowledge, the potential use of anxiety disorder biomarkers is only a suggestion, and this topic requires in-depth research [115]. Moreover, it is possible that different biomarkers are associated with a group of symptoms and not with a specific diagnosis [177]. The most promising biomarkers are listed in the table below (Table 1). The saliva biomarkers described in this review only show their potential application in practice. Presumably, promising markers, such as structural differences in the amygdala and hippocampus, although they proved to be highly reliable [29,82], are not commonly used in diagnostics for practical and economic reasons [177]. To enable the use of biomarkers and their dissemination, we would need simple and economically advantageous biomarkers [15]. Moreover, the identification of biomarkers is based on the observation that a specific biomarker is only detected in affected patients [177]. However, due to the overlapping pathophysiological symptoms of psychiatric disorders, biomarkers may be common to various psychiatric disorders, which may lead to an interpretation bias, thus to the lack of availability of highly specific biomarkers [178,179]. Even though some of the biomarkers listed for panic disorders had high sensitivity, they did not show sufficient specificity to distinguish PD from other psychiatric disorders [82]. Most of the suggested biological markers described above (e.g., structural brain morphology, lower plasma 5-HT concentration, increased/decreased cortisol secretion, PWD, and RDW) can be used to differentiate patients with panic disorders from healthy subjects they can not be used to differentiate from patients with other psychiatric disorders, such as other anxiety disorders, schizophrenia, or mood disorders with hyperactive HPA [149,178]. It should also be noted that biomarkers are influenced by environmental and lifestyle factors such as stress, physical activity, comorbidities, and medications [177].

Overall, it is highly unlikely that a single common biomarker for anxiety disorders can be established. However, even though the diagnosis of anxiety disorders is still largely based on clinical symptoms, biomarkers could be a valuable tool to help identify individual patients with the disorder, improve treatment fit, and predict treatment responses. Such use of a biomarker is already common in other medical fields for various diseases such as asthma and rheumatoid arthritis [180,181], but detection of such a marker in psychiatric disease will be one of the most difficult tasks that researchers will ever face [182]. Identifying beneficial biomarkers can help diagnose and classify a group of psychiatric disorders. Further exploration of biomarkers in psychiatry should focus efforts on numerous clinical populations, with the harmonization of biomarker specificity and their importance in clinical practice [177].

## Figures and Tables

**Table 1:** Potential biomarkers of anxiety disorders and their variability.

Salivary Biomarker	Blood Biomarker	Cerebrospinal Fluid	Neuroimaging Marker
Cortisol ↓ [33,36] (PD)	Serotonin ↑ [68] (OCD)	5-HIAA ↑ [119] (PD)	Amygdala activity ↑ [151,153] (Stress)
sIgA ↓ [38,40] (Stress)	BDNF ↓ [76] (PD, GAD)	CCK ↓ [133] (PD)	ACC ↑ [155] (Stress)
Melatonin ↓ [49] (PTSD)	NPS ↑ [78,79] (GAD)	Oxytocin ↓ [129] (Stress)	Insula ↑ [158] (PTSD)
sAA ↑ [53] (Stress)	Cortisol ↓ [87,89] (PD)	Ghrelin ↑ [139] (Sress)	dACC ↑ [161,163] (PTSD)
FGF-2 ↓ [67] (Stress)	IM: IL-1, IL-6, CRP ↑ [94,95] (PTSD, GAD)		
	HDL-C ↓ [109] (OCD)		

↑: increased concentration/activity; ↓: decrease concentration/activity. sIgA: Immunoglobulin A; sAA: Alpha-amylase; CgA: Chromogranin A; FGF-2: Fibroblast Growth Factor 2; BDNF: brain-derived serum neurotrophic factor; NPS: Neuropeptide S; IM: inflammatory markers; 5-HIAA: 5-Hydroxyindoleacetic acid; ACC: subgenual anterior cingulate cortex; dACC: dorsal ACC; PD: Panic disorder; PTSD: post-traumatic stress disorder; GAD: generalized anxiety disorder; OCD: obsessive-compulsive disorder.

## Data Availability

Data sharing not applicable No new data were created or analyzed in this study. Data sharing is not applicable to this article.
